# Rac1 signaling in the establishment of the fucoid algal body plan

**DOI:** 10.3389/fpls.2014.00690

**Published:** 2014-12-10

**Authors:** Whitney E. Hable

**Affiliations:** Department of Biology, University of Massachusetts Dartmouth, Dartmouth, MA, USA

**Keywords:** actin, cell polarization, morphogenesis, Rac1 GTP binding protein, *Silvetia*, *Fucus*

## Abstract

Fucoid zygotes use environmental vectors, including sunlight, to initiate a growth axis a few hours after fertilization. The first division is then transversely oriented by the growth axis, producing daughter cells of distinct fates. The tip growing rhizoid cell gives rise to the holdfast, anchoring the alga to the intertidal substratum, while the opposite thallus cell mainly generates the photosynthetic and reproductive stipe and fronds. Elaboration of this simple growth axis thus establishes the basic body plan of the adult; and elucidating the mechanisms responsible for formation of the growth axis is paramount to understanding fucoid morphogenesis. Recent studies have culminated in a model whereby sunlight, and perhaps other environmental cues, activate the signaling protein Rac1 at the rhizoid pole. Here it sets in motion nucleation of a patch of actin filaments that in turn, targets ions, proteins, and cellular processes to the future growth site. At germination, Rac1 initiates morphogenesis by inducing transformation of the patch of actin filaments to a structure that delivers vesicles to the growing tip, and a few hours later orients the spindle and cytokinetic plate.

## INTRODUCTION

For many multicellular organisms, establishment of a basic body plan occurs early in development and is a critical process in morphogenesis, organizing cell division, expansion and differentiation to specify, and properly place tissues and organs. Often, an initial axis of polarity, where molecules or other subcellular components become asymmetrically distributed, is generated in the single-celled egg or zygote. The cues that orient this axis can be inherited from maternal tissues or perceived from external sources. For example, in the fruit fly *Drosophila melanogaster*, polarity is specified in the unfertilized egg by asymmetrically deposited mRNAs and proteins from maternal nurse cells (Huynh and St Johnston, 2004). In contrast, polarity in the nematode *Caenorhabditis elegans* is not established in the egg, rather at fertilization sperm entry specifies the posterior region of the developing embryo (Goldstein and Hird, 1996).

**FIGURE 1:**
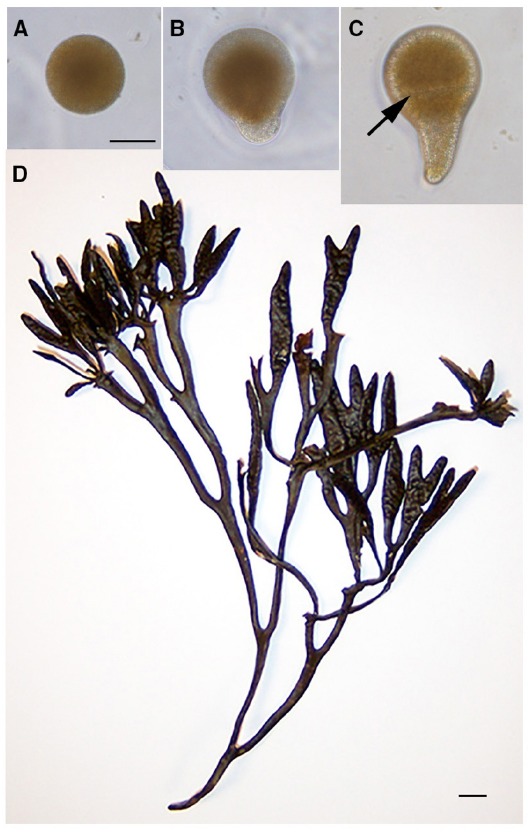
**A simple growth axis establishes the basic body plan of fucoid algae.** The unfertilized zygote **(A)** is radially symmetric. A few hours later tip growth (germination) begins, first observed as a local swelling at the rhizoid pole **(B)**. The rhizoid–thallus growth axis orients the transverse first division (black arrow; **C**) generating daughter cells of distinct fates. The rounded thallus cell contributes mainly to the stipe and fronds of the adult **(D)**, while the tip-growing rhizoid cell largely give rise to the holdfast (not shown). Scale bar in **(A)** is 50 *μ* and also serves for **(B,C)**. Scale bar in **(D)** is 0.5 cm.

Fucoid brown algae, in the stramenopile lineage, establish a basic body plan from a simple growth axis that is initiated a few hours after fertilization (AF; Figure 1). During this time, the radially symmetric zygote gives way to localized growth at the rhizoid pole (Figures 1A,B). This growth axis orients the first division, which is transverse and asymmetric (Figure 1C), producing daughter rhizoid and thallus cells. Continued growth and division of the tip growing rhizoid cell generates a file of cells that will largely give rise to the holdfast (Kropf, 1992), attaching the alga to the rocky substratum in the intertidal zone. Meanwhile, the thallus cell proliferates in three dimensions producing a ball of cells that will mainly generate the photosynthetic and reproductive stipe and fronds (Figure 1D; Kropf, 1992). For nearly 100 years, there has been much interest in the mechanisms specifying the rhizoid–thallus axis, as it initiates morphogenesis of the adult structure.

Species of *Fucus* and *Silvetia*, within the Fucaceae, inhabit the coastal intertidal zone of most of the northern hemisphere (Serrao et al., 1999), surviving extremes of temperature and salinity, as well as changes in tides. Just a few hours AF, non-buoyant zygotes secrete a polyphenolic adhesive that firmly attaches them to the rocky substratum (Vreeland et al., 1998). This initial adhesion prevents displacement by subsequent tides and also allows the zygotes to remain fixed in space with respect to vectorial information in their surroundings. Ungerminated zygotes are responsive to a host of environmental signals that can dictate the position of the rhizoid pole. But even before this, at fertilization the position of sperm entry specifies a labile default rhizoid pole, ensuring that zygotes will initiate rhizoid growth and have means to establish a body plan no matter the environmental circumstances (Hable and Kropf, 2000). In nature, the absence of environmental cues is probably rare, and the fertilization-induced growth axis is typically overridden by directional signals including light, temperature, ions (Weisenseel, 1979), chemicals secreted by a nearby zygote (Hurd, 1920; Whitaker and Lowrance, 1940) nutrients, and bioluminescence from nearby algal thalli (Jaffe, 2005). Sunlight is likely the most common cue, with rhodopsin-like molecules (Robinson et al., 1998) and aureochrome photoreceptors (Takahashi et al., 2007) perceiving light in the UV and blue wavelengths. Rhizoid growth subsequently occurs on the shaded hemisphere (Figure 1B).

**FIGURE 2:**
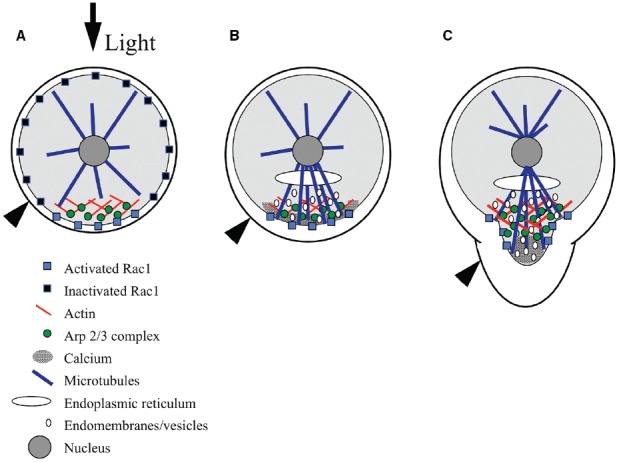
**Model for signaling during polarity establishment. (A)** Rac1 distribution is initially symmetric at the plasma membrane, and is selectively activated at the rhizoid pole in response to environmental cues, like light (black arrow). The Arp2/3 complex is localized by Rac1, where it nucleates a patch of filamentous actin. **(B)** The F-actin patch targets a calcium gradient, Rac1, microtubules, endomembrane vesicles, and adhesive secretion toward the rhizoid pole. Microtubules localize the endoplasmic reticulum and contribute to the polar delivery of adhesive-bearing vesicles. **(C)** At germination, Rac1 directs Arp2/3 complex-dependent actin nucleation in the subapex of the growing rhizoid and microtubules carry vesicles toward the tip; vesicles fuse with the plasma membrane in regions devoid of actin but enriched with calcium, at the very tip. Black arrowhead indicates surface of adhesive.

Many studies have shown that establishment and maintenance of the rhizoid–thallus growth axis is dependent on filamentous actin (F-actin) arrays (Quatrano, 1973; Hable and Kropf, 1998; Bisgrove and Kropf, 2001; Hable et al., 2003). In the unfertilized egg, actin filaments are distributed uniformly throughout the cell cortex (Kropf et al., 1989). During fertilization, a patch of F-actin forms at the rhizoid pole (Figure 2A); this patch is observed by 30 min AF, the earliest time point that can be experimentally observed (Hable and Kropf, 2000). If no additional polarizing cue is detected by the zygote, rhizoid growth commences from the site of the sperm-induced F-actin patch several hours later (Figure 2B). However, prior to rhizoid growth, both the rhizoid pole and the F-actin patch are labile, and if the zygote perceives relevant environmental cues, the growth axis is reoriented and the sperm-induced F-actin patch is replaced by a new patch of F-actin at the new rhizoid pole (Alessa and Kropf, 1999). Formation of the new F-actin patch most likely occurs by polymerization of a new array, rather than repositioning of the sperm-induced F-actin patch, as treatments that either stabilize or depolymerize actin filaments block reorientation of the growth axis (Alessa and Kropf, 1999; Hable et al., 2003). Moreover, very briefly as repolarization is occurring, two small F-actin patches can be observed, one at the former rhizoid pole and one at the new rhizoid pole. These F-actin arrays are hypothesized to represent the transitional depolymerizing and newly polymerizing patches, respectively (Alessa and Kropf, 1999).

In ungerminated zygotes, the F-actin patch appears to be responsible for positioning a suite of molecules and cellular processes at the rhizoid pole in preparation for growth (Figure 2B). Following F-actin patch formation, the next detectable asymmetry is a gradient of intracellular Ca^2+^, highest at the rhizoid pole (Pu et al., 2000; Pu and Robinson, 2003). Formation of the gradient is blocked by actin-depolymerizing drugs (Pu et al., 2000). The Ca^2+^ gradient may not be functionally required until later, as treatments that disrupt the gradient do not prevent the perception of a light cue (Hable et al., 2001). The F-actin-dependent Ca^2+^ gradient is independent of earlier Ca^2+^ fluxes that are associated with karyogamy and sperm pronuclear migration (Bothwell et al., 2008). Microtubules are nucleated from all sides of the nucleus prior to germination; however, the F-actin patch directs an enrichment of microtubules extending to the rhizoid cortex (Peters and Kropf, 2010). The rhizoid microtubules localize the ER and guide vesicle trafficking (Peters and Kropf, 2010), enhancing polarized endomembrane activity (Hadley et al., 2006), and polar secretion of adhesive (Hable and Kropf, 1998). Although microtubules are not necessary for polar growth, they clearly help control rhizoid shape as destabilizers like oryzalin result in a short, blunt tip (Peters and Kropf, 2010).

As germination begins, the F-actin patch transforms to a structure sometimes observed as a collar at the subapex of the tip (Alessa and Kropf, 1999; Pu et al., 2000) and other times as a cone that extends from the nucleus to the rhizoid subapex (Figure 2C; Hable et al., 2003; Hable and Kropf, 2005). It is unclear whether differences in the precise organization of actin are artifacts of the preparations, or if this reflects different arrays that are normally both present in the rhizoid. There is precedence for two distinct actin arrays in the pollen tube tips of lily (Lovy-Wheeler et al., 2005) and tobacco (Fu et al., 2001). In any cell preparation, the tip of the rhizoid is devoid of F-actin, which may itself be an artifact of the difficulty in preserving F-actin in growing tips, as in pollen tubes (Hepler et al., 2001). Alternatively, the absence of F-actin may constitute a permissive zone at the rhizoid apex for vesicle fusion with the plasma membrane. As the tip lengthens, the F-actin structure also elongates, maintaining a clear zone at the apex, again where new membrane insertion is occurring (Hable and Kropf, 2005). The actin array closest to the tip is dynamic; its establishment and maintenance is inhibited by pharmacological compounds that either depolymerize or stabilize actin filaments. Moreover, these treatments indicate that growth itself is dependent on dynamic actin structures (Quatrano, 1973; Hable et al., 2003).

Germination is also accompanied by even greater enrichment of molecules and processes in the tip, presumably initiating tip-localized growth (Figure 2C). The Ca^2+^ gradient is more pronounced by this time (Berger and Brownlee, 1993; Taylor et al., 1996), and treatments that disrupt it abolish growth (Roberts et al., 1993). The enhanced Ca^2+^ gradient and a newly apparent gradient of reactive oxygen species (ROS) appear to be part of a reciprocal amplification loop. The two gradients coincide spatially, and inhibitor studies disrupting one or the other demonstrate that they are interdependent (Coelho et al., 2008). Similarly, polar adhesive secretion (Hable and Kropf, 1998) and polar endomembrane (Fowler et al., 2004) activity are enriched in growing rhizoids. Evidence suggests that F-actin in the growing rhizoid continues to target calcium, adhesive, endomembrane cycling and microtubules, as these processes are also dependent on an intact actin cytoskeleton (Hable and Kropf, 1998; Pu et al., 2000; Hadley et al., 2006). Whether the tip-actin array also controls the ROS gradient has yet to be tested.

## FORMATION OF DYNAMIC F-ACTIN ARRAYS

Formation of the F-actin patch is a common response to at least three different cues (default sperm entry point, light, neighbor) and may be universal to all relevant environmental signals (Alessa and Kropf, 1999; Hable and Kropf, 2000). Less is known of the mechanisms regulating the actin arrays during polarity establishment and maintenance, and how a zygote might coordinate multiple polarizing signals. Recent studies implicate a signaling pathway in which Rac1, a small monomeric GTP binding protein, localizes the Actin related protein 2/3 complex (Arp2/3 complex), which in turn, nucleates actin filaments at the rhizoid pole (Hable and Kropf, 2005; Hable et al., 2008; Muzzy and Hable, 2013).

The Arp2/3 complex is one of several eukaryotic actin nucleators, and is composed of seven highly conserved protein subunits. First identified in *Acanthamoeba castellanii* (Machesky et al., 1994), it was originally shown to nucleate actin assembly in lamellipodial extension and in the rocket-like tails that propel movement of some intracellular pathogens (Borisy and Svitkina, 2000; Cooper and Schafer, 2000). In *Silvetia compressa*, it appears to be responsible for forming and remodeling F-actin arrays at the rhizoid pole. Immunolocalization of Arp2 and F-actin demonstrate colocalization of these proteins at the rhizoid pole of young zygotes in response to cues from sperm, light or a neighboring zygote (Figures 2A,B; Hable and Kropf, 2005). In germinated zygotes, Arp2 colocalizes with the F-actin cone or collar in the growing tip (Figure 2C; Hable and Kropf, 2005). Moreover, when the direction of tip growth is altered by a change in the direction of light (negative phototropism), growth in the new direction is preceded by relocalization of both Arp2 and F-actin (Hable and Kropf, 2005).

In many cell types, such as migrating animal cells, the Arp2/3 complex is activated through a Rac1-dependent signaling pathway. Rac1 is one of many GTP binding proteins in the Ras superfamily and Rho subfamily (reviewed in Bustelo et al., 2007; Heasman and Ridley, 2008; Mucha et al., 2011). Ras proteins are ubiquitous in eukaryotes, and highly conserved. These small proteins behave as molecular switches, cycling between GTP and GDP bound forms. When associated with GTP, they activate downstream effectors, in many cases leading to the Arp2/3 complex-dependent nucleation of actin arrays. When GTP is hydrolyzed, the GDP-bound form is inactive. Guanine nucleotide exchange factors, or GEFs, activate Ras proteins by inducing release of GDP and association with GTP. GTPase activating proteins, or GAPs, enhance the intrinsic GTPase activity within Ras proteins, switching them “off.” Further, guanine dissociation inhibitors (GDIs) regulate membrane association of some Ras superfamily members by masking a post-translationally added lipid tail (Seabra, 1998).

In the fucoid algae, Rac1 was first identified in a cDNA library from *Fucus distichus*, and was found to partially rescue a Rho family mutant (*cdc*42) in budding yeast (Fowler et al., 2004). This result is consistent with a functional overlap with the CDC42 protein, which induces Arp2/3 complex-dependent nucleation of actin patches in yeast (Moreau et al., 1996). Further, immunolocalization data demonstrate that the protein (FdRac1) localizes to the growing tip of *F. distichus* zygotes and embryos (Fowler et al., 2004). More recently, Rac1 has been immunologically identified in *S. compressa*. Although the *S. compressa* gene has yet to be identified, as the genome has not been sequenced, a peptide antibody developed against a consensus between FdRac1 and the single Rac1 gene in *Ectocarpus siliculosus* (Cock et al., 2010; in the same division and class) detects a single protein of the predicted size (21 kDa) in *S. compressa* (Muzzy and Hable, 2013). Because the peptide antigen was unique to Rac1 and not present in other monomeric GTPases, the antibody is unlikely to be detecting anything other than Rac1. In the first few hours AF, Rac1 is uniformly localized to the zygote cortex, perhaps tethered to the membrane (Figure 2A). A few hours later, around the time that adhesive secretion and endomembrane activity become polarized, Rac1 transitions to a patch that colocalizes with F-actin at the rhizoid pole (Figure 2B). As tip growth occurs, Rac1 forms a diffuse collar that overlaps with F-actin in the rhizoid subapex (Figure 2C; Muzzy and Hable, 2013).

Formation of the F-actin patch and maintenance of an F-actin cone after germination both require Rac1 activity. The membrane permeable compound NSC23766 (NSC), has been shown to specifically inhibit Rac1 activity by blocking the GEF recognition groove without affecting other Rho family GTPases (Gao et al., 2004). In young *S. compressa* zygotes, NSC disrupts F-actin patch formation in a dose-dependent manner, resulting in patches that are diffuse, delocalized, or absent (Muzzy and Hable, 2013). Additionally, cellular processes dependent on this actin array are inhibited; NSC delocalizes and reduces adhesive secretion, delocalizes endomembrane cycling, and delays germination (Hable et al., 2008). When germinated zygotes are treated, NSC distorts the subapical F-actin and overlapping Arp2 structure; these cytoskeletal arrays are still observed near the nucleus, but are conspicuously absent from the tips (Hable et al., 2008). Further, NSC alters rhizoid morphology producing greatly expanded, swollen tips and reduced tip growth rate (Hable et al., 2008). These data are consistent with a process in which Rac1 targets the nucleation of actin filaments at the rhizoid pole, and then as growth proceeds, expands actin nucleation into the tip. Decreased Rac1 activity in turn prevents continued actin nucleation and tip growth becomes delocalized.

Rac1 membrane association may also be important for regulation of its activity. Both FdRac1 (Fowler et al., 2004) and the *E. siliculosus* Rac1 sequence (Cock et al., 2010) contain a C-terminal CVIS domain which specifies the post-translational addition of a farnesyl moiety to the protein. In *S. compressa*, inhibiting farnesylation with the membrane permeable chemical manumycin A abolishes Rac1 signal in both young ungerminated and older tip growing zygotes, suggesting that Rac1 is normally tethered to the rhizoid membrane with its lipid tail. Moreover, cellular processes normally targeted by F-actin, such as polarized endomembranes, polar adhesive secretion and germination are inhibited by manumycin A treatment. Indeed F-actin localization itself is eliminated in treated zygotes (Muzzy and Hable, 2013). Because manumycin A targets the enzyme farnesyl transferase, and not specifically the farnesylation of Rac1, it cannot be ruled out that a different farnesylated protein other than Rac1 is regulating these processes.

A model for Rac1 signaling during morphogenesis is emerging whereby sunlight, and perhaps other environmental cues, trigger asymmetric activation of Rac1 at the rhizoid pole of the zygote (Figure 2; Muzzy and Hable, 2013). Rac1 activation then leads to Arp2/3 complex-dependent polymerization of actin in a patch that prepares the tip for growth. As the recently fertilized egg assesses its environment for polarizing cues, Rac1 is distributed diffusely throughout the cortex, where its selective activation would lead to rapid nucleation of the actin patch (Figure 2A; Muzzy and Hable, 2013). It is critical that fucoid zygotes establish their growth axes quickly, in order to prevent being washed out of the rocky intertidal zone in the next tide cycle. The initially diffuse Rac1 distribution is thus an advantage because it allows for rapid positioning of the rhizoid pole at any site on the zygote surface. Based on other eukaryotes, activation of the Arp2/3 complex is probably not direct, and may be through the Scar/WAVE complex, as homologs of this complex are present the genome (Cock et al., 2010) of the related alga, *E. siliculosus*. The Arp2/3 complex, in turn, nucleates actin filaments to form a patch at the rhizoid pole (Hable and Kropf, 2005). The F-actin patch targets the localization of a gradient of calcium, Rac1 protein, microtubules, adhesive secretion and endomembranes to the future growth site (Hable and Kropf, 1998; Pu and Robinson, 2003; Hadley et al., 2006; Peters and Kropf, 2010; Muzzy and Hable, 2013). At germination, Rac1 and the Arp2/3 complex transform this patch to a cone or collar that participates in vesicle delivery to the growing tip (Hable et al., 2008; Muzzy and Hable, 2013). A few hours later, the growth axis orients the spindle and cytokinetic plate transversely to establish the asymmetric division that crudely sets up the body axis (Bisgrove and Kropf, 2001).

## FUTURE DIRECTIONS

Many gaps in Rac1 signaling to polarity remain to be filled. What proteins mediate activation of the Arp2/3 complex? Is Rac1 involved in the transmission of *all* polarizing cues? Given that a zygote almost always chooses one site for rhizoid outgrowth, how does a zygote respond to two or more cues that specify different rhizoid poles? How is Rac1 itself activated at the rhizoid pole? Rho family small GTPases, especially Rac1 and Rho of plants (ROPs), are involved in polarizing a wide range of cell types, and understanding the spatial regulation of Rac1 activity is of great interest. On the one hand, activation and inactivation by GEFs and GAPs contribute; understanding how these regulators are spatially arranged within a cell is ongoing. On the other hand, RhoGDIs play an important role in controlling the availability of small GTPases. The literature pertaining to Rac1 and ROP regulation is vast and therefore not covered in this mini-review; however, these topics are reviewed in depth by Bloch and Yalovsky (2013), Bustelo et al. (2012), Craddock et al. (2012), Ngok et al. (2014), Thompson (2013), and Yang and Lavagi (2012). Without fully sequenced genomes available for the fucoid algae, we will continue to rely on other related genomes, like that of *E. siliculosus*. However, a recent breakthrough in the use of RNAi to knock down gene expression in *Fucus serratus* (Farnham et al., 2013) has greatly expanded the possibilities for addressing these and other questions. Gene-silencing has long been a much-needed missing tool that will complement the powerful microscopy, physiology and chemical inhibition in the fucoid algae. As our understanding of the signaling pathways responsible for establishment of the rhizoid–thallus grows, the mechanisms guiding the basic body plan of the alga are beginning to be revealed.

### Conflict of Interest Statement

The author declares that the research was conducted in the absence of any commercial or financial relationships that could be construed as a potential conflict of interest.
